# Chromosome-End Knockoff Strategy to Reshape Alkaloid Profiles of a Fungal Endophyte

**DOI:** 10.1534/g3.116.029686

**Published:** 2016-06-20

**Authors:** Simona Florea, Timothy D. Phillips, Daniel G. Panaccione, Mark L. Farman, Christopher L. Schardl

**Affiliations:** * Department of Plant Pathology, University of Kentucky, Lexington, Kentucky 40546; † Department of Plant and Soil Sciences, University of Kentucky, Lexington, Kentucky 40546; ‡ Division of Plant and Soil Sciences, West Virginia University, Morgantown, West Virginia 26506

**Keywords:** chanoclavine, Clavicipitaceae, ergotryptamine, ergovaline

## Abstract

Molecular genetic techniques to precisely eliminate genes in asexual filamentous fungi require the introduction of a marker gene into the target genome. We developed a novel strategy to eliminate genes or gene clusters located in subterminal regions of chromosomes, and then eliminate the marker gene and vector backbone used in the transformation procedure. Because many toxin gene clusters are subterminal, this method is particularly suited to generating nontoxic fungal strains. We tested this technique on *Epichloë coenophiala*, a seed-transmissible symbiotic fungus (endophyte) of the important forage grass, tall fescue (*Lolium arundinaceum*). The endophyte is necessary for maximal productivity and sustainability of this grass but can produce ergot alkaloids such as ergovaline, which are toxic to livestock. The genome sequence of *E. coenophiala* strain e19 revealed two paralogous ergot alkaloid biosynthesis gene clusters, designated *EAS*1 and *EAS*2. *EAS*1 was apparently subterminal, and the *lpsB* copy in *EAS*2 had a frame-shift mutation. We designed a vector with a fungal-active hygromycin phosphotransferase gene (*hph*), an *lpsA*1 gene fragment for homologous recombination at the telomere-distal end of *EAS*1, and a telomere repeat array positioned to drive spontaneous loss of *hph* and other vector sequences, and to stabilize the new chromosome end. We transformed *E. coenophiala* with this vector, then selected “knockoff” endophyte strains, confirmed by genome sequencing to lack 162 kb of a chromosome end including most of *EAS*1, and also to lack vector sequences. These ∆*EAS*1 knockoff strains produced no detectable ergovaline, whereas complementation with functional *lpsB* restored ergovaline production.

Tall fescue (*Lolium arundinaceum = Schedonorus arundinaceus = Festuca arundinacea*) is a perennial cool season grass widely used in the United States as a forage crop and for conservation and amenity purposes, due to its persistence, tolerance of drought and other stresses, and resistance to pests and diseases (Malinowski and Belesky 2000, 2006; Schardl *et al.* 2004, 2013a; Timper *et al.* 2005; Nagabhyru *et al.* 2013; Joost 2009), and excellent biomass yield. Remarkably, these exceptional qualities are not strictly plant properties, but rather are significantly enhanced by the symbiotic, seed-transmitted fungus (endophyte), *Epichloë coenophiala* (= *Neotyphodium coenophialum*), which colonizes the above-ground parts of the host without causing harm to the plant (Schardl *et al.* 2004). Furthermore, *E. coenophiala* cannot spread between neighboring plants, because the only means by which it transmits is by colonization of the seeds. Only plants symbiotic with the endophyte produce seeds with the endophyte, and they do so with extremely high efficiency (Siegel *et al.* 1984). Fitness benefits conferred by *E. coenophiala* to tall fescue plants include enhanced tillering, enhanced root growth, improved mineral uptake, increased drought tolerance (Malinowski and Belesky 2000; Elbersen and West 1996; Nagabhyru *et al.* 2013), and increased resistance to nematodes (Elmi *et al.* 2000; Timper *et al.* 2005), diseases, and insect pests (Schardl *et al.* 2004, , 2013a). Like other *Epichloë* species, *E. coenophiala* produces a diverse array of alkaloids, specialized (secondary) metabolites that are not required by the fungus for growth and development, but instead protect against vertebrate or invertebrate herbivores (di Menna *et al.* 2012; Tanaka *et al.* 2005; Schardl *et al.* 2013a). Alkaloids such as lolines and peramine provide important protection from insects and perhaps nematodes (Bacetty *et al.* 2009a,b). However, “common toxic endophyte” (CTE) strains of *E. coenophiala* also produce ergot alkaloids that, due to their effects on livestock, reduce the endophyte benefits for pasture and forage production (Watson *et al.* 2004; Hopkins *et al.* 2010; Schardl *et al.* 2013a).

According to their complexity, ergot alkaloids can be classified in three groups: clavine alkaloids, lysergic acid and its simple amides, and the notoriously toxic ergopeptines (Gerhards *et al.* 2014; Robinson and Panaccione 2015; Young *et al.* 2015). Their biosynthesis (Figure 1A) proceeds through clavines to lysergic acid, then to the ergopeptines, such as ergovaline produced by *E. coenophiala* CTE strains. Ergot alkaloid synthesis (*EAS*) genes that encode the biosynthetic enzymes are clustered and located near the chromosome ends in sequenced genomes of *Epichloë* species (Schardl *et al.* 2013b,c). Those ends are protected by telomeres comprised of CCCTAA tandem repeat units (Farman 2011). In all, 11 *EAS* genes determine the pathway to ergovaline. However, *E. coenophiala* CTE strains have duplicate sets of ergot alkaloid biosynthesis genes because *E. coenophiala* is a near triploid hybrid fungus, with genomes from three ancestral species (Schardl *et al.* 2013c). Two of those ancestors have contributed *EAS* loci, the third has contributed a loline biosynthesis locus, and all three have contributed peramine biosynthesis loci (Schardl *et al.* 2013c).

**Figure 1:**
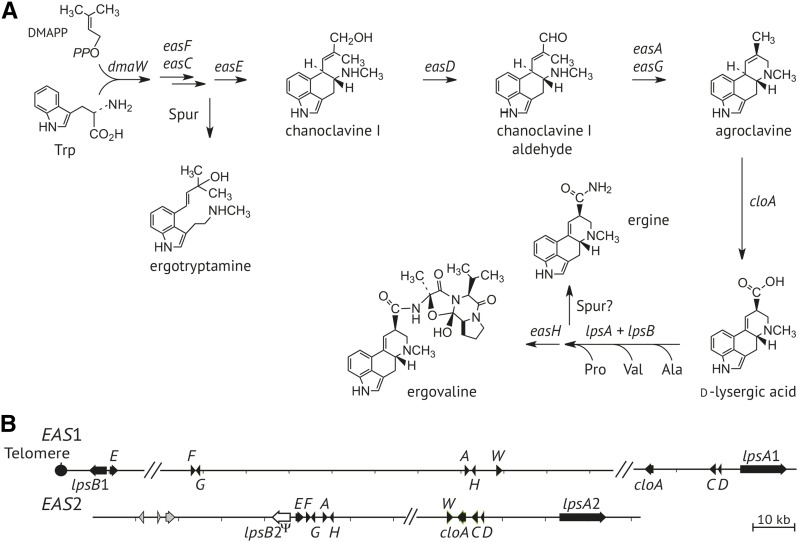
Ergot alkaloid pathway and genes. (A) Summary of the ergot alkaloid biosynthesis pathway showing major intermediates and products. Arrows are labeled with genes that direct those steps in the pathway. Details for the biosynthesis of spur products, ergotryptamine and ergine, are unknown. The L-amino acids are labeled by their standard three-letter codes. DMAPP, dimethylallyl diphosphate. (B) Ergot alkaloid gene clusters *EAS*1 and *EAS*2 in *E. coenophiala* e19. Names of *eas* genes and *dmaW* are abbreviated to their final capital letters. The *lpsB*2 pseudogene, which has an inactivating frame-shift mutation, is shown as an open-box arrow. Genome sequencing confirmed linkage of *lpsB*1 and *easE*1 to a telomere at the position shown. Hash marks indicate gaps in the assembly, but the putative genes orders shown are similar to those in the genome assembly of *E. coenophiala* strain e4163.

Intensive surveys of tall fescue in Europe and North Africa (Christensen *et al.* 1993; Marlatt *et al.* 1997) have identified some Moroccan ecotypes that lack ergot alkaloids, and certain nontoxic endophytes (NTE) have been cultured from those ecotypes and used to replace the CTE in order to produce novel cultivars. As expected, livestock performance on the novel cultivars is significantly better than on cultivars with their original CTE, and not significantly different than on tall fescue lacking endophyte (Watson *et al.* 2004; Hopkins *et al.* 2010; Parish *et al.* 2003). However, the NTE strains currently used for novel cultivars are derived from very different tall fescue ecotypes (summer dormant, Moroccan) than the northern European ecotypes from which the summer active cultivars are derived, and in which the NTE are now being deployed. For that reason it is unsurprising that problems have been reported with the NTE strains in those cultivars. Some have exhibited less stability than the CTE strains (Bouton *et al.* 2002) or appear less effective against root-parasitic nematodes (Timper *et al.* 2005), a potentially important limitation to productivity and drought tolerance in the southeastern United States (Elmi *et al.* 2000). The reasons that Moroccan endophytes exhibit inconsistent antinematode activity are unknown, but conceivably relate to lower production of loline alkaloids, which are translocated to roots (Schardl *et al.* 2007) and can affect nematodes (Bacetty *et al.* 2009a,b). Thus, although the search for existing NTE strains has been a commercial and agricultural success, they may not be the optimal choices for mixing and matching plant and symbiont strains for some US pasturelands.

Here we present a novel approach to generate nontoxic endophyte strains, based on the tendency for toxin genes to be located near chromosome ends (Schardl *et al.* 2013b). This approach was also designed to abolish all exogenous genes including the selectable marker used in endophyte transformation. We used this approach to eliminate the telomere-associated *EAS*1 gene cluster from the genome of a European *E. coenophiala* ecotype, and confirmed that the resulting ∆*EAS*1 strains lacked transgenes. These strains lack ergovaline, and may be suitable, therefore, for tall fescue pastures and forage.

## Materials and Methods

### Biological materials

The wild-type *E. coenophiala* strain e19 (=ATCC 90664) was isolated from tall fescue (*L. arundinaceum*) cv. Kentucky 31 (Tsai *et al.* 1992), and *E. coenophiala* strain e4163 was isolated from a tetraploid *L. arundinaceum* plant (PI # 422777 from Western Regional Plant Production Station, Pullman, WA). The *E. coenophiala* strain e7135 (Florea *et al.* 2009), was derived from e19 by replacing the ergot alkaloid biosynthesis gene *dmaW*2 with a hygromycin B-resistance gene (*hph*), followed by elimination of *hph*. These and other strains generated in this study were cultured and maintained as described in Florea *et al.* (2015). The *E. coenophiala* strains generated in this study, and their respective genotypes, were designated e7479 ∆*EAS*1, e7480 ∆*EAS*1, and e7481 ∆*EAS*1 derived from e19, e7575 ∆*dmaW*2 ∆*EAS*1 derived from e7135 ∆*dmaW*2, and the *lpsB*-complemented strain e7605 ∆*EAS*1 *lpsB* generated from e7479 ∆*EAS*1. Single-spore isolates of each strain are designated e7479-1, e7479-2, and so forth.

Because *E. coenophiala* is not contagious, and cannot move into a plant except by vertical transmission in seeds, new plant lineages symbiotic with endophyte strains are established by artificial inoculations (Latch and Christensen 1985). Endophyte-free seeds of tall fescue elite breeding line KYFA0601 were germinated and the seedlings inoculated with *E. coenophiala* strains by the method of Chung *et al.* (1997). Each strain was introduced into 100 seedlings, of which ca. 80% survived after inoculation. The inoculated seedlings were planted in soil and allowed to grow in the greenhouse to produce multiple tillers. The bases of two vegetative tillers from each plant were assayed for endophyte presence by tissue-print immunoblot with antiserum raised against *E. coenophiala* protein (An *et al.* 1993). The resulting plants that were symbiotic with each strain were numbered as follows: number 6105 had e7479 ∆*EAS*1, number 6106 had e7480 ∆*EAS*1, number 6107 had wild-type e19, number 6212 had e7575 ∆*dmaW*2 ∆*EAS*1, and number 6221 had e7605 ∆*EAS*1 *lpsB*. For each alkaloid and gene expression analysis we used five independently inoculated plants as replicates.

### Molecular methods

Fungal DNA was isolated from fresh mycelium using ZR Fungal/Bacterial DNA MiniPrep kit (Zymo Research, Irvine, CA), or using Geno/Grinder 2000 (SPEX CertiPrep, Metuchen, NJ) and DNeasy 96 Plant Kit (Qiagen, Valencia, CA). Plasmid DNA was isolated from bacterial cultures using the ZR Plasmid Miniprep-Classic kit (Zymo Research, Irvine, CA). The mRNA was isolated from plant material using RNeasy Plant Mini Kit (Qiagen). PCR screens were performed using AmpliTaq Gold, and AmpliTaq Gold PCR buffer provided by the manufacturer (Applied Biosystems, Foster City, CA). For vector construction, the PCR amplifications were performed with Phusion Hot Start High-Fidelity DNA Polymerase (Thermo Scientific, Ratastie, Vantaa, Finland) with Phusion HF buffer (with 1.5 mM MgCl_2_) from the manufacturer. The temperature conditions were 98° for 3 min, followed by 35 cycles of 98° for 10 sec, 62° for 10 sec, and 72° for 7 min, then a final 5 min incubation at 72°. The oligonucleotides used in this study were from Integrated DNA Technologies (Coralville, IA) and are listed in Table 1.

**Table 1:** Primers used in this study

Primer Name	Sequence
lpsA1SpeI(f)	GGGACTAGTTAAGAAGCGCTTACGCCGTTCC
lpsA1MluI(r)	CACACGCGTAGCTGTCGTATGAAGGCACGAT
polylinkerDdeI	/5Phos/TAAGCTCGAGGCCATGATGGCCTTTAAAGTCTACGTACTCA
polylinkerSpeI	/5Phos/CTAGTGAGTACGTAGACTTTAAAGGCCATCATGGCCTCGAGC
dmaWe19copy2(+)-1d	AGAAACAGACAGGGCTATTC
dmaWe19copy2-(−)-5u	CTCGCCGGCATGCGTCAAAA
dmaw1(f)	TTATTGGATGAAACCTTAGCTAGTTGG
dmaWe19(-)-10	CTCGCCGGCATGCGTCAAAT
144lpsBDraI(f2)	CACTTTAAACCTAATGCACTACACTAAGACCCC
144lpsB(r)	AATCTGGCCAACATGGTTCCCATG
215hphlpsB(f)	GCTTGACAAACGCACCAAGTTATCG
215lpsBhph(r)	TGTACACCACTTCAACGAGGCTTG
hph.3d	CGAAGTTATCTCGACGGTATCG
hph.3u	TCGGCGAGTACTTCTACACA
RTq-E.c.easE(f)	TCCTTGCCACCAAGGCAGATTG
RTq-E.c.easE(r)	ACATTGTCCACGGCAAGCCCTC
RTq-E.c.easA(f)	CGTGCGGATAATGAAGGCGTCC
RTq-E.c.easA(r)	CGATGAGAAATCCATTGGCACCG
RTq-E.c.easC(f)	GGCATGGCAGTCAAGTTCTTCAC
RTq-E.c.easC(r)	ACATTGGCTGTCCAAGTAGGGT
RTq-E.c.easF(f)	CCCAGAACTTTCGTCATGTCCG
RTq-E.c.easF(r)	ATCCCGTCCAGTGGCGGAAGTA
RTq-E.c.easG(f)	TTGCCAAGACTCTCCATGAGAT
RTq-E.c.easG(r)	ACCACGTCGGTCTTAATACAGCG
oligoscreen(f)	GATGGCCTTTAAAGTCTACGTACTC
lpsAoligo(r)	ATATCATGGCAACATTCAGCGCAC

### Genome analysis

Genome sequencing and assembly was performed at the Advanced Genetic Technologies Center (AGTC) of the University of Kentucky, and all genome assemblies were done with Newbler 2.8 (Roche Diagnostics/454 Life Sciences Corporation). The genome of the wild-type *E. coenophiala* strain e19 was sequenced by a combination of pyrosequencing (Roche) of sheared DNA fragments and Sanger sequencing of fosmid-cloned ends (Schardl *et al.* 2013b). Pyrosequencing reads totaled 6,008,281, giving 2,219,715,337 nt of data. These included 84,573 ditags, of which 49,817 were in the same scaffold (average distance = 2.7 kb ± 0.8 kb). The 19,471 sequenced fosmid ends (totaling 14,229,733 bp) gave 7143 read-pairs, of which 3104 assembled in the same scaffold at 36,371 ± 9093 bp distance (range = 18,185–54,556 bp). In total, 2,201,579,615 nt assembled into 95,835,721 bp in 15,240 contigs, and the total inferred lengths of the 5640 scaffolds was 99.6 Mb (including unsequenced gaps), with N50 = 39,442 bp in 567 scaffolds (∼22-fold coverage).

The genome of e7479 ∆*EAS*1 was sequenced by a combination of Ion Torrent PGM (Life Technologies) and 454 pyrosequencing (Roche Diagnostics) to give 2,790,692 Ion Torrent reads totaling 454,053,270 nt, and 855,760 extended pyrosequencing reads totaling 628,200,560 nt. Assuming a 99.6 Mb genome size (from the scaffold assembly of e19), this was 10.9-fold coverage. Assembly with Newbler 2.8 gave 46,534 contigs totaling 87,313,881 bp, of which 32,285 large contigs (≥500 bp) totaled 83,439,153 with N50 = 3871 bp.

The genome of e7480 ∆*EAS*1 was sequenced on the MiSeq platform (Illumina, San Diego, CA) to give 22,358,620 reads at 250 cycles, totaling 4,711,677,366 high-quality bases. This was an estimated 47-fold coverage. Assembly with CLC Genome Workbench 8.0 (CLC Bio LLC, Waltham, MA), with default parameters, gave 41,794 contigs totaling 70,103,076 bp, N50 = 9061 bp. Approximately equal base representations of A, T, G, and C in the total assembly indicated that the AT-rich intergenic regions were underrepresented in this assembly.

### Plasmid constructs

**Figure 2:**
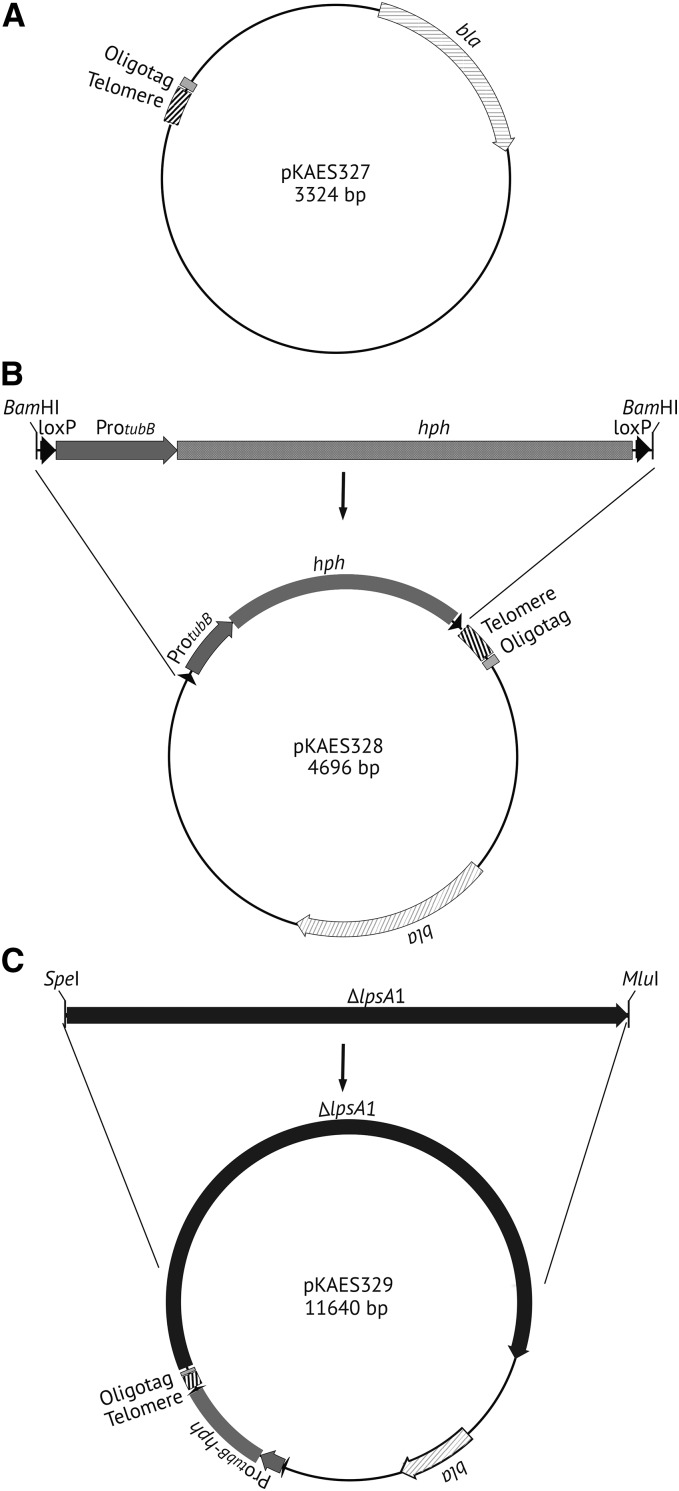
Plasmids constructed in this study. (A) A telomere repeat array and adjacent oligotag were introduced into pKAES215 to produce pKAES327, which has a β-lactamase gene for selection in bacteria. (B) A loxP-flanked hygromycin phosphotransferase gene (*hph*), downstream of the promoter of *E. typhina tubB* (gene for β-tubulin), was introduced into pKAES327 to give pKAES328. The loxP sites allow for Cre-mediated excision of the marker, a procedure that was not required in this study. (C) A 6944 bp fragment of the *E. coenophiala* e19 *lpsA*1 gene was introduced into pKAES328 to give pKAES329.

A previously cloned telomere repeat array from *E. festucae* consisting of 26 tandem repeats of CCCTAA (Farman 2011) was excised with *Sau3A*I and *Dde*I. In addition, a 45 bp synthetic “oligotag” (5′-TAAGCTCGAGGCCATGATGGCCTTTAAAGTCTACGTACTCACTAG-3′) was derived from two complementary oligonucleotides (polylinkerDdeI and polylinkerSpeI) that were annealed to provide restriction endonuclease cleavage sites *Dde*I, *Dra*I, *Rsa*I, *SnaBI*, and *Spe*I. The oligotag was cleaved with *Dde*I and *Spe*I, and ligated to the 3′ side of the telomere repeat array and the correspondingly digested vector pKAES215 (Pan *et al.* 2014) to give plasmid pKAES327 (Figure 2A). Then a hygromycin B-resistance gene cassette Pro*_tubB_-hph* (hereafter designated *hph*) (Spiering *et al.* 2008) was ligated into the *BamH*I site of pKAES327 at the 5′ side of the telomere repeat array to give pKAES328 (Figure 2B). This plasmid can be further modified by introducing a target sequence from near a chromosome end into the oligotag, such that homologous recombination will generate a “knockoff” of the genes between the target sequence and the telomere. In this study, plasmid pKAES329 (Figure 2C) was designed to knock off *EAS*1 in *E. coenophiala*, and was generated by PCR-amplifying with primers lpsA1SpeI(f) and lpsA1MluI(r) a 6944 bp fragment of the *E. coenophiala* e19 *lpsA*1 gene, digesting the PCR product with *Spe*I and *Mlu*I, and ligating it into the *Spe*I and *Mlu*I sites in the oligotag of correspondingly digested pKAES328.

To construct the *lpsB*-complementation plasmid pKAES362, pKAES215 was digested with *Spe*I, end-repaired using End-it DNA End Repair kit (Epicentre, Madison, WI) and then digested with *Xba*I. The digested vector was ligated, using the Fast-Link DNA ligation kit (Epicentre), to a fragment containing the *lpsB* gene and its native promoter (from *E. festucae* × *typhina* strain Lp1), which had been generated by PCR with primers 144lpsBDraI(f2) and 144lpsB(r) and then digested with *Xba*I and *Dra*I.

### Fungal transformation

*Epichloë coenophiala* isolates were grown in potato dextrose broth and the protoplasts were prepared and transformed using the polyethylene glycol method as described previously (Panaccione *et al.* 2001; Florea *et al.* 2009), except that, prior to transformation, the plasmid DNA was incubated for 30 min with 10 μg of Lipofectin Transfection Reagent (Life Technologies). Protoplasts of *E. coenophiala* e19 and e7135 were transformed with 6–10 μg of pKAES329 DNA linearized with *Mlu*I. The complementation transformation was performed with 8 μg of pKAES362 linearized with *Xba*I. The protoplasts were then suspended in 7 ml CRM-low (complete regeneration medium containing low melting agarose from Seakem LE, FMC Bioproduct, Rockland, ME) (Panaccione *et al.* 2001), and poured over 20 ml complete regeneration medium (CRM) plates containing hygromycin B (Calbiochem, San Diego, CA) to give a final concentration of 50 μg/ml. The transformation plates were incubated at 21° for 4–5 wk. For the chromosome-end knockoff experiment the fungal transformants were transferred onto potato dextrose agar (PDA) without hygromycin B (nonselective medium) for sporulation, and then single-spore isolated on nonselective medium. For the complementation experiment the transformants were maintained on PDA containing hygromycin B.

### Screening of the knockoff and complementation transformants

To identify putative Δ*EAS*1 knockoffs the fungal transformants were screened by PCR as follows. DNA was extracted with the DNeasy 96 Plant Kit (Qiagen, Valencia, CA) and screened by PCR with primers specific for *dmaW*1 [dmaW1(f) and dmaWe19(-)-10] and *dmaW*2 (dmaWe19copy2.1d and dmaWe19copy2.5u). All of the putative knockoffs were also screened for the presence or absence of *hph* by PCR with the primer pair hph.3d and hph.3u. For complementation of the Δ*EAS*1 knockoff strain the transformants were screened for integration of the *lpsB*-containing plasmid by PCR with the primer pair 215hphlpsB(f) and 215lpsBhph(r). The PCR reactions were carried out in 25 μl reaction mixtures with 5–10 ng DNA template, 200 μM each dNTP, 0.2 μM each primer, 2.5 units AmpliTaq Gold, and AmpliTaq Gold PCR buffer with MgCl_2_ (1.5 mM final conc.) provided by the manufacturer (Applied Biosystems, Foster City, CA), in a model 2720 Thermal Cycler (Applied Biosystems). The temperature regime was as follows: 9 min at 95°, 35 cycles of 94° for 30 sec, annealing temperature (61° for *dmaW*2, 57° for *lpsB-hph*, 59° for *dmaW*1 and *hph*) for 35 sec, 72° for 2 min, and then a final 7 min incubation at 72°.

### Antibiotic sensitivity tests

Mycelium of each putative Δ*EAS*1 knockoff strain was ground in 500 μl sterile water and aliquots were spread on PDA with and without hygromycin B (50 μg/ml) in wells of Falcon 6-well plates (Becton Dickinson and Co., Franklin Lakes, NJ). The plates were incubated for 4 wk at 21°.

### Ergot and loline alkaloid analyses

Alkaloid profiles were determined from 1-yr-old plants symbiotic with the *E. coenophiala* strains, and five independently inoculated plants were analyzed for each strain. Ergot alkaloids were extracted from 20 to 50 mg of freeze-dried tall fescue pseudostems and analyzed by high-pressure liquid chromatography (HPLC) as previously described (Panaccione *et al.* 2012) based on the method of Spiering *et al.* (2002). Loline alkaloids were extracted from 50 mg of freeze-dried pseudostems and analyzed by GC-MS as described by Blankenship *et al.* (2001).

### Gene expression assays

For each symbiotic association the total RNA was isolated from five individual first generation plants using RNeasy Plant Mini Kit (Qiagen, Valencia, CA) according to the manufacturer’s instructions, and quantified using a Qubit Fluorometer (Invitrogen, Waltham, MA). Any DNA contaminating the RNA samples was removed by incubating with 20 U RNase-free DNase I (Promega Corp. Madison, WI) at 37° for 20 min. The cDNA was synthesized from 1 μg RNA using the High Capacity cDNA Reverse Transcription kit (Applied Biosystems, Waltham, MA) and oligo(dT) primer. The relative quantification PCR reactions (qPCR) were run on the ABI PRISM 7900HT instrument. Gene expression assays were performed using primer pairs for the *EAS* genes *dmaW*, *easA*, *easC*, *easD*, *easE*, *easF*, *easG*, and *cloA* (Table 1) and Power SYBR Green PCR Master Mix (Applied Biosystems), with 15 ng cDNA template per 25 μl reaction, and 0.4 μM each primer. The housekeeping gene *tefA* (encoding translation elongation factor 1-α) was used as reference gene for normalization. Cycle threshold values (*C_T_*) were calculated with SDS 2.3 software, with the default baseline setting of 3–15 cycles. To control overplate variations, probes for the target genes and the reference gene, *tefA*, were arrayed on each plate and all the reactions were run in triplicate. The relative gene expression levels were calculated using the ΔΔ*C_T_* (Livak and Schmittgen 2001) method and converted into fold-difference (2^- ΔΔ^*^CT^*) relative to the median of each target gene.

### Seed transmission tests

The plants of elite breeding line KYFA0601 symbiotic with *E. coenophiala* e7479-1 ∆*EAS*1 and 7480-1 ∆*EAS*1 were grown in the greenhouse for 1 yr, then planted and vernalized in the field. The seeds were harvested from each plant and stored separately at 20°. For each of the two knockoff strains, 10 plants were checked for endophyte transmission by analysis of eight seeds each as follows. DNA was extracted using the DNeasy 96 Plant Kit (Qiagen) according to the manufacturer’s instructions, except that plates with arrayed seeds were immersed in liquid nitrogen for 30 sec immediately prior to maceration with the Geno/Grinder 2000. The PCR screen for the presence of the oligotag linked to remnant *lpsA*1 was performed with primers oligoscreen(f) and lpsAoligo(r) and the following program: 9 min at 95°, 35 cycles of 94° for 30 sec, 59° for 35 sec, 72° for 1 min, and then a final 7 min incubation at 72°.

### Data availability

Genbank reference numbers: KC989569.1, KC989570.1, KC989607.1, KC989608.1, KC989609.1, KC989610.1, and KC989611.1.

## Results

### Identification of EAS gene clusters in E. coenophiala

The genome sequence assembly for wild-type *E. coenophiala* strain e19 included two copies each of the 11 *EAS* genes known to be required for ergovaline production (Young *et al.* 2015), although the assembly did not contain the *EAS* clusters entirely within individual scaffolds. As is typical of *EAS* clusters in *Epichloë* species (Schardl *et al.* 2013b), regions flanking and between *EAS* genes were primarily composed of very AT-rich repeats, which probably interfered with complete assemblies of the clusters. However, the previously reported genome sequence of another wild-type *E. coenophiala* strain, e4163, had one scaffold with its entire *EAS*1 cluster (GenBank KC989569.1) and another with its entire *EAS*2 cluster (GenBank KC989570.1) (Schardl *et al.* 2013c). The cluster with genes most similar to those of *E. festucae* was designated *EAS*2, the other was designated *EAS*1, and the orthologous copies in e19 were identified by identity or near identity of their nucleotide sequences to those of the corresponding cluster in e4163. Assuming that the gene arrangements in e19 are similar to those in e4163, tentative maps were generated and are given in Figure 1B. The only *EAS* genes in e19 that lacked orthologs in e4163 were *lps*B1 and *easE*1, which assembled together on an 18,217 bp single-contig scaffold of the e19 assembly (GenBank accession KC989609.1). The reverse-complement of that accession terminated in a canonical telomere repeat array (5′-TAGGGTTAGGGTTAGGGTTAGGGTTAGGGTTAGGG-3′) downstream of *lpsB*1. Therefore, if all *EAS*1 genes are linked in e19, then the *EAS*1 cluster is located at a chromosome end.

### Identification of putative ΔEAS1 chromosome-end knockoff strains

Transformation plasmid pKAES329 was constructed with a 6944 bp segment of *lpsA*1 sequence to target homologous integration, an *hph* selectable marker for hygromycin B resistance (Spiering *et al.* 2008), and a telomere repeat array between them to eventually separate *hph* and the rest of the vector backbone from the *lpsA*1 sequence (Figure 2). Between the telomere sequence and *lpsA*1 target sequence was a 45 bp synthetic “oligotag,” (5′-TAAGCTCGAGGCCATGATGGCCTTTAAAGTCTACGTACTCACTAG-3′), to facilitate definitive identification of the genomic changes expected in ∆*EAS*1 knockoff strains. After transformation, colonies were selected on regeneration plates with hygromycin B, and then 192 colonies were tested by PCR for *dmaW*1 and *dmaW*2. Three colonies were identified as negative for *dmaW*1 and positive for *dmaW*2, as would be expected if the linearized pKAES329 had integrated by homologous recombination at *lpsA*1, causing loss of the corresponding chromosome end. These three transformants were designated e7479 ∆*EAS*1, e7480 ∆*EAS*1, and e7481 ∆*EAS*1. A similar transformation was conducted on *E. coenophiala* e7135 ∆*dmaW*2, a strain in which part of *dmaW*2 had been replaced by *hph*, which in turn had been removed by the action of Cre recombinase (Florea *et al.* 2009). A PCR screen of 67 transformants revealed one putative chromosome-end knockoff strain, designated e7575 ∆*dmaW*2 ∆*EAS*1.

### Tests for spontaneous losses of vector sequences

**Figure 3:**
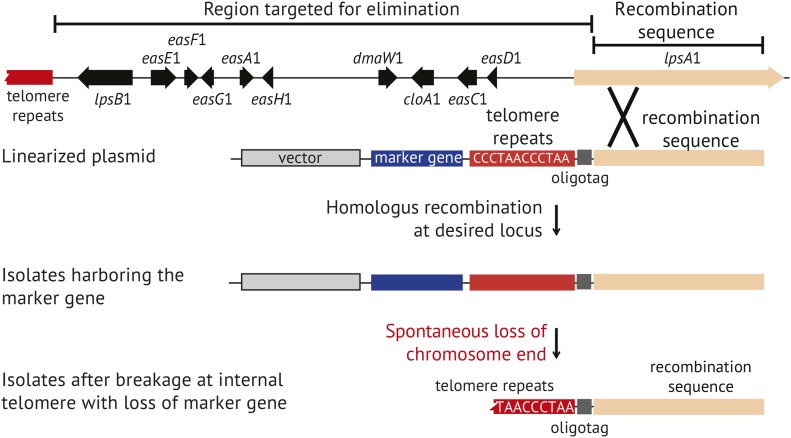
Schematic representation of the chromosome knockoff strategy for elimination of toxin genes. The plasmid is linearized by *Mlu*I digestion on the telomere-distal side of the recombination sequence, and then introduced into fungal protoplasts. Transformants are selected based on the selectable marker, such as the *hph* gene for hygromycin B resistance. If the plasmid has integrated by homologous recombination, genes between the recombination sequence and the chromosome end should be lost in the absence of selection because they are no longer linked to a centromere-containing chromosome. Subsequent breakage at the introduced telomere repeat array results in loss of the selectable marker and vector backbone, and the remaining telomere stabilizes the new chromosome end. Components of this diagram and intergenic regions are not drawn to scale.

**Figure 4:**
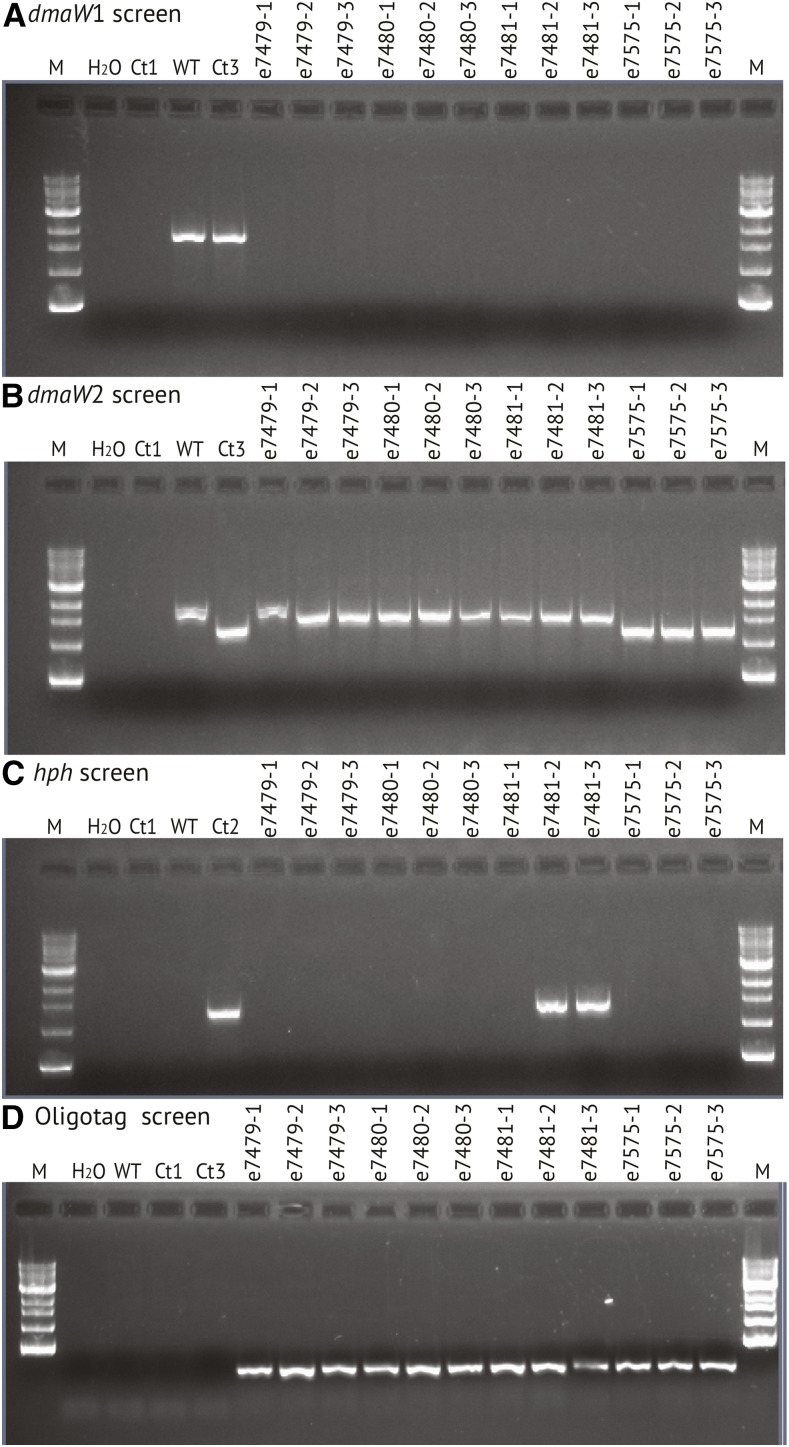
PCR tests of single-spore isolates of putative ∆*EAS*1-knockoff transformants. (A) Results of PCR with primers specific for *dmaW*1 from the *EAS*1 cluster. (B) Results of PCR with primers specific for *dmaW*2 from the *EAS*2 cluster. (C) Results of PCR tests for *hph*. (D) Results of PCR tests for the introduced oligotag linked to the *lpsA*1 remnant. Controls (see Florea *et al.* 2009) are: H_2_O = no template PCR control; WT = e19, with both *dmaW*1 and *dmaW*2; Ct1 = *Epichloë uncinata* e167, which lacks all *EAS* genes; Ct2 = *E. coenophiala* e7133, a derivative of e19 that possesses *dmaW*1 but has an *hph* cassette in place of a partial deletion in *dmaW*2; Ct3 = e7135, which is derived from e7133 by Cre-mediated elimination of the *hph* cassette.

Each of three putative knockoff transformants was transferred onto PDA without hygromycin B (nonselective medium), and then single-spore isolated on nonselective medium. For each, three single-spore isolates were then randomly chosen and designated e7479-1, e7479-2, e7479-3, and so forth. The single-spore isolates were retested by PCR for the *dmaW* copies, and the results confirmed loss of *dmaW*1 and retention of *dmaW*2 (Figure 4, A and B). Spontaneous breakage at the internal telomere repeat was expected to lead to loss of *hph* and the vector backbone with all foreign DNA except the 45 bp oligotag (Figure 3). To check for such events, the single-spore isolates were screened by PCR for *hph* (Figure 4C). For two of the transformants (e7479 ∆*EAS*1 and e7480 ∆*EAS*1) all single-spore isolates tested negative for *hph*, but for the third transformant (e7481 ∆*EAS*1) two out of three single-spore isolates tested positive for *hph*. All three unselected single-spore isolates from e7575 ∆*dmaW*2 ∆*EAS*1 tested negative for *hph*. These strains were also confirmed to contain the oligotag, based on PCR with one primer sequence contained within the oligotag and the other primer sequence contained within the *lpsA*1 remnant (Figure 4D).

To confirm loss of *hph*, the single-spore isolates were tested for sensitivity to hygromycin B. All isolates that tested negative for *hph* by PCR were sensitive to the antibiotic, whereas the two *hph*-positive single-spore isolates from e7481 ∆*EAS*1 retained the ability to survive on selective medium. Similarly, all spores derived from e7575 ∆*dmaW*2 ∆*EAS*1 failed to grow on medium with hygromycin B. The two transformants that showed no retention of *hph* or hygromycin B resistance were considered to be confirmed ∆*EAS*1 knockoff derivatives of e19, and a single-spore isolate of each (e7479-1 ∆*EAS*1 and e7480-1 ∆*EAS*1) was maintained for further study.

### Genome sequencing of knockoff strains

Inspection of assembled genome sequences of e7479-1 ∆*EAS*1 and e7480-1 ∆*EAS*1 by BLASTn revealed the *EAS*2-cluster genes, but none of the *EAS*1-cluster genes, as expected for *EAS*1 chromosome-end knockoffs. Furthermore, there were no other sequences from the transformation vector except the expected *lpsA*1 remnant and the 45 bp noncoding oligotag sequence. The e7480-1 ∆*EAS*1 genome assembly included a 181 bp contig with sequence 5′-TAACCCTAACCCTAACCCTAAGCTCGAGGCCATGATGGCCTTTAAAGTCTACGTACTCACTAGTTAAGAAGCGCTTACGCCGTTCCACTTGTGCCTTTGACTGGATGATGGATACAGATAGTAACTAACCGTGGACAGTATGATATTATTATGCACGTGGATTCCAAACAACAATGTTACC-3′. This has three telomere repeats (repeat unit TAACCC) followed by the oligotag at positions 19–63, and *lpsA*1 sequence at positions 65–181. Similarly, the e7479-1 ∆*EAS*1 assembly included a 228 bp contig with telomere sequence at positions 1–106, the oligotag at positions 107–151, and partial *lpsA*1 sequence at positions 153–228. Thus, both strains had assembled contigs with the sequences expected for the truncated chromosome end.

### Alkaloid profiles in symbio

Tall fescue seedlings were inoculated with single-spore isolates of e7479 ∆*EAS*1, e7480 ∆*EAS*1, and e7575 ∆*dmaW*2 ∆*EAS*1 to establish systemic symbioses. In addition, e7479 ∆*EAS*1 was complemented with wild-type *lpsB* to give strain e7605 ∆*EAS*1 *lpsB+*, and that was also introduced into plants, as was wild-type e19. Ergovaline and ergine were undetected in the plants symbiotic with the ∆*EAS*1-knockoff strains, but those plants had the early pathway spur product, ergotryptamine (Ryan *et al.* 2015), levels averaging 87 and 100 nmol/g dry weight, which exceeded the amounts of total ergot alkaloids usually observed in plants symbiotic with the wild-type strain (Table 2). Chanoclavine I was also observed at higher concentrations than in the plants with the wild-type strain. Additionally, plants with the *lpsB*-complemented strain accumulated chanoclavine I, ergine, and ergovaline to concentrations similar to those of plants with the wild-type strain, and ergotryptamine to concentrations similar to those of plants with the uncomplemented ∆*EAS*1-knockoff strains. No ergot alkaloids were detected in plants symbiotic with e7575 ∆*dmaW*2 ∆*EAS*1.

**Table 2:** Ergot alkaloid profiles in tall fescue pseudostems with endophytic *E. coenophiala* strains

Endophyte Strain	Endophyte Genotype	Ergovaline (nmol/g)	Ergine (nmol/g)	Ergotryptamine (nmol/g)	Chanoclavine I (nmol/g)
e19	WT	12.6 ± 4.1	2.7 ± 1.6	2.7 ± 2.1	3.0 ± 2.2
e7479-1	∆*EAS*1	0.0	0.0	86.7 ± 74	5.6 ± 3.7
e7480-1	∆*EAS*1	0.0	0.0	100.0 ± 41	5.3 ± 1.2
e7605	∆*EAS*1 *lpsB+*	12.1 ± 6.8	2.1 ± 1.3	112.7 ± 75	2.5 ± 1.3

Alkaloid concentrations are averages ± SEM for five replicates.

Plants symbiotic with the knockoff strains had loline alkaloid profiles similar to those with the wild-type strain e19 (data not shown).

### Gene expression profiling of ∆EAS1 knockoff strains

**Figure 5:**
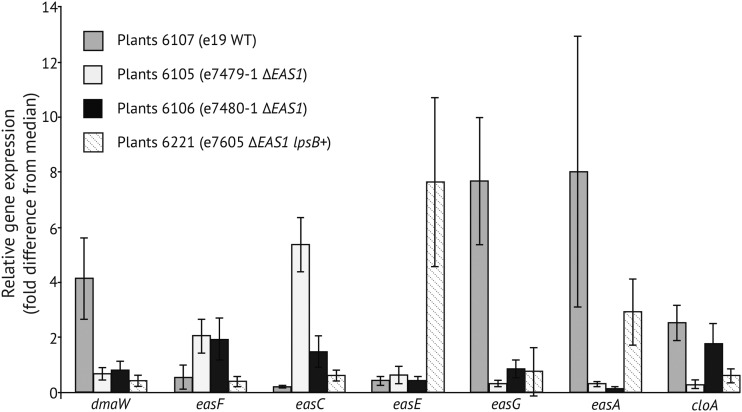
Relative expression of genes for early steps of the ergot alkaloid pathway. Each plant number series had the endophyte isolate with the genotype indicated in parentheses. For each series, five infected plants were randomly selected and analyzed as replicates. Gene expression as measured by RT-qPCR is indicated as fold-difference from the median for each gene in the experiment. Error bars are SEM.

*In symbio* gene expression profiling indicated several changes in Δ*EAS*1 knockoff strains compared to wild-type e19. A lower expression of *dmaW*, *easG*, and *easA* was observed in the knockoff strains, whereas *easF* and *easC* were expressed at higher levels compared to the wild-type e19 (Figure 5). Expression of *easD* appeared unchanged in e7480 ∆*EAS*1 compared to the wild type (data not shown). The complemented strain (e7605 ∆*EAS*1 *lpsB+*) had *dmaW*, *easC*, *easG*, and *cloA* expression levels similar to those of its parental knockoff strain, e7479 ∆*EAS*1, whereas its *easF* and *easA* expression levels were similar to those of wild-type e19 (Figure 5). The *easE* expression level in the *lpsB*-complemented strain exceeded that of both the wild-type and knockoff strains, whereas *dmaW* and *easG* expression remained low in the complemented strain.

### Symbiotic stability of the knockoff strains

After seedling inoculation with the endophyte strains the tall fescue plants were grown in the greenhouse for ∼1 yr, then planted in the field where they vernalized over winter and then set seeds. In seed tests, strong PCR-positive results indicated that at least 96% had *E. coenophiala*, and the other 4% were considered nondefinitive because they gave less PCR product. Thus, endophyte seed transmission for the first seed harvest was high (≥96% infection rate) for both e7479-1 ∆*EAS*1 and e7480-1 ∆*EAS*1 knockoff strains. The ergot alkaloid profile of samples derived from these seeds was similar to the profile of vegetative tissues derived from plants associated with the knockoff strains, except that the seeds had much less ergotryptamine relative to the levels measured in pseudostems (Table 3).

**Table 3:** Ergot alkaloid profile and concentrations in first generation seeds

Plant Series*^a^*	Endophyte Genotype	Ergovaline (nmol/g)	Ergine (nmol/g)	Ergotryptamine (nmol/g)	Chanoclavine (nmol/g)
6105	e7479-1 ∆*EAS*1	0.0	0.0	9.0	0.8
6105	e7479-1 ∆*EAS*1	0.0	0.0	8.7	0.8
6106	e7480-1 ∆*EAS*1	0.0	0.0	9.3	0.8
6106	e7480-1 ∆*EAS*1	0.0	0.0	6.4	0.7
6107	e19 WT	9.4	7.9	1.1	1.4
6107	e19 WT	10.3	7.9	1.5	1.8

a Each sample was a pool of seeds from five plants of the same series. Two samples from each series were analyzed.

## Discussion

Many years of research have established that CTE *E. coenophiala* provides numerous fitness enhancements to tall fescue cultivars used throughout much of the United States (Malinowski and Belesky 2006; Hopkins *et al.* 2010; Schardl *et al.* 2013a). Most such cultivars and the naturalized populations of tall fescue in North America, Australia, and New Zealand have northern European origins, and the strict vertical transmission and ubiquity of CTE strains in tall fescue throughout northern Europe (Ekanayake *et al.* 2012; Takach and Young 2014; Young *et al.* 2014) suggest that the hosts and endophytes are closely coadapted. We rationalized, therefore, that surgically eliminating toxin-production genes from a CTE strain would probably generate nontoxic strains that retain the vast majority and magnitude of benefits to the plant. However, currently available techniques for such genetic manipulations in asexual fungi necessarily leave transgenes. (In contrast, for sexual species crossing strategies can eliminate such transgenes.) In a previous study (Florea *et al.* 2009) we employed marker-exchange mutagenesis with a loxP-flanked *hph* selection marker, screened for the desired gene replacement, and then transiently transformed the mutant with a Cre recombinase gene to eliminate *hph*. Though effective, this approach was far more intensive and expensive than the alternative we present here, whereby we targeted telomere-associated clusters of genes. By sequencing genomes of *E. coenophiala* e4163 (Schardl *et al.* 2013c) and e19 (this work), we determined that the *EAS*1 cluster was subterminal, and also that *lpsB*2, a key ergovaline-synthesis gene in the e19 *EAS*2 cluster, was probably inactive. Then we devised a strategy to generate chromosome-end knockoff mutants lacking the *EAS*1 cluster. To do so required a vector that contained a telomere repeat array to stabilize the resulting chromosome end, but we positioned that sequence such that the vector, including *hph*, would be lost upon breakage at the introduced telomere. The main risk was that *hph* might be insufficiently stable in the transformants for initial selection. In fact, we recovered hygromycin B-resistant transformants, and those with the target-site integration were identified based on marker instability after single-spore isolation on nonselective medium. Using this strategy, we eliminated ∼162 kb of the *EAS*1 cluster from the genome of strain e19, and similarly knocked off *EAS*1 from strain e7135, a ∆*dmaW*2-knockout produced previously. As expected, the e19 ∆*EAS*1 strains produced no ergovaline, and the ∆*dmaW* ∆*EAS*1 strain produced no ergot alkaloids.

Since sequences of the *EAS*2-cluster genes suggested that all could be functional except *lpsB*2, we expected that plants with the ∆*EAS*1 knockoff strains would accumulate lysergic acid as previously shown for an *lpsA* knockout strain of a perennial ryegrass endophyte (Panaccione *et al.* 2001, 2003). However, the ∆*EAS*1 strains produced no detectable lysergic acid or even the intermediate tetracyclic clavines. Instead, the ergot alkaloid profiles were dominated by ergotryptamine and chanoclavine I, similar to the profile previously observed when the four early pathway genes (*dmaW*, *easF*, *easC*, and *easE*) from *Neosartorya fumigata* were introduced into the ergot alkaloid nonproducer, *Emericella nidulans* (Ryan *et al.* 2013). Ergotryptamine is a spur product produced in *Em. nidulans* expressing *dmaW*, *easF*, and *easC*, as well as several unmodified *Epichloë* species including *E. coenophiala* (Ryan *et al.* 2015), whereas chanoclavine I is an intermediate in the pathway to lysergic acid. Since ergovaline production was restored by complementation with a functional *lpsB*, the other genes in the *EAS*2 clusters are apparently functional. However, the relatively high level of ergotryptamine in the ∆*EAS*1 strains as well as the *lpsB*-complemented strain suggests that the EasE2 protein may not be fully active. Sequence comparisons of e19 *easE*2 with *easE* in other *Epichloë* species known to produce ergot alkaloids indicated a nonsynonymous mutation at codon 230, giving a serine in place of the otherwise conserved proline. If this P230S mutation affected EasE2 function, the production of chanoclavine I by the ∆*EAS*1 strains indicated that EasE2 had at least some activity (unless another unknown enzyme provided complementary activity). Furthermore, the alkaloid profile of the complemented strain, with levels of ergovaline and ergine similar to wild-type e19, suggested more complex dynamics than just a bottleneck at the EasE step. Clearly, there is more to learn about ergot alkaloid pathway regulation in *E. coenophiala*.

*In symbio EAS* gene expression in wild-type, ∆*EAS*1-knockoff, and *lpsB*-complemented strains was highly variable, and the differences between strains were not always as expected; nor were they indicative of the changes in alkaloid profiles. Compared to wild type, the ∆*EAS*1-knockoff had dramatically lower expression of *dmaW*, *easA*, and *easG*, in keeping with loss of one of two gene copies; but other *EAS* genes showed similar or higher expression levels. Also, even though only *lpsB* was introduced for complementation, several other *EAS* genes also exhibited altered expression in the complemented strain. Nevertheless, even with expression changes that sometimes exceeded eightfold in key genes (*e.g.*, *easE*), the alkaloid profiles – and particularly the elevated ergotryptamine levels in plants with ∆*EAS*1 and ∆*EAS*1 *lpsB+* strains – are not easily correlated with gene expression changes.

The reason for developing and deploying our technology to generate nontransgenic, yet genetically altered strains, is to avoid risk either real or perceived associated with transgenic organisms. Existing methods for surgical genetic manipulation of asexual fungi require introduction of marker genes derived from other organisms. Such exogenous (“foreign”) genes pose public and regulatory concerns, especially since these endophytes are to be deployed in cultivars that are meant to persist in pastures for decades. Once planted, it would be difficult or impossible to recall the plant with its modified endophyte. Therefore, it is essential for us to address up front the regulatory and public concerns associated with genetically modified organisms. The nature of our knockoff strains were reviewed by the Animal and Plant Health Inspection Service (APHIS) and determined not to fall under their regulation. We note that the alkaloid profiles of plants with the knockoff strains are similar to those of some naturally occurring grass-*Epichloë* species symbiota that accumulate chanoclavine as an end product (Schardl *et al.* 2013c). In rats, chanoclavine I exhibits no appreciable effect on dopamine receptors (Watanabe *et al.* 1987) and prolactin levels (Cassady *et al.* 1974). Ergotryptamine is a newly identified ergot alkaloid (Ryan *et al.* 2015) for which the biological activities are yet to be determined.

Small animal and livestock feeding studies are now needed to determine the effects, if any, of the simple ergot alkaloids associated with the ∆*EAS*1 knockoff strains. Also needed are plant performance studies in the field, during which the specific alterations in the genome, including the 45 bp oligotag, will facilitate monitoring strain persistence in plant lines and field plots, and possible movement in agroecosystems.
